# Supplementary material for: Atacformer: A transformer-based foundation model for analysis and interpretation of ATAC-seq data
Source: bioRxiv. 2025 Nov 4:2025.11.03.685753. Preprint. [Version 1] doi: 10.1101/2025.11.03.685753 (PMC12637716; doi:10.1101/2025.11.03.685753)
Supplement: 1 [file NIHPP2025.11.03.685753v1-supplement-1.pdf]

bioRxiv preprint doi: https://doi.org/10.1101/2025.11.03.685753; this version posted November 4, 2025. The copyright holder for this preprint (which was not certified by peer review) is the author/funder, who has granted bioRxiv a license to display the preprint in perpetuity. It is made available under aCC-BY 4.0 International license.

Supplemental material

Supplementary Table S1. *Overview of datasets used in the pre-training of Atacformer.*

Dataset	Tissue	Disease state	No. cells	GSE/Link	Author
Human single-cell atlas	Atlas	Healthy	615,998	GSE184462	Ren 2021
Brain 107k	Brain	Healthy	107,057	GSE168408	Lister 2023
Atlas of tonsil	Tonsil	Healthy + Disease	70,775	-	Massoni-Badosa 2025
Luecken2021	Blood	Healthy	69,249	-	Luecken2021
Parkinson’s 65k	Brain	Disease	65,589	GSE148434	Jung 2023
Muscle 23k	Muscle	Disease	23,593	GSE174376	Dyer 2022
Kidney 22k	Kidney	Healthy	22,772	-	10X
Cornea atlas	Eye	Healthy	1,209	GSE155683	Lako 2021

Supplementary Table S2. *Overview of multiome datasets used in the CRAFT fine-tuning process.*

Dataset	Tissue	Disease state	No. cells	Source
Brain3k Multiome	Brain	Healthy	3,233	10X
Kidney22k Multiome	Kidney	Healthy	22,772	10X
PBMC10k Multiome	Blood	Healthy	10,970	10X

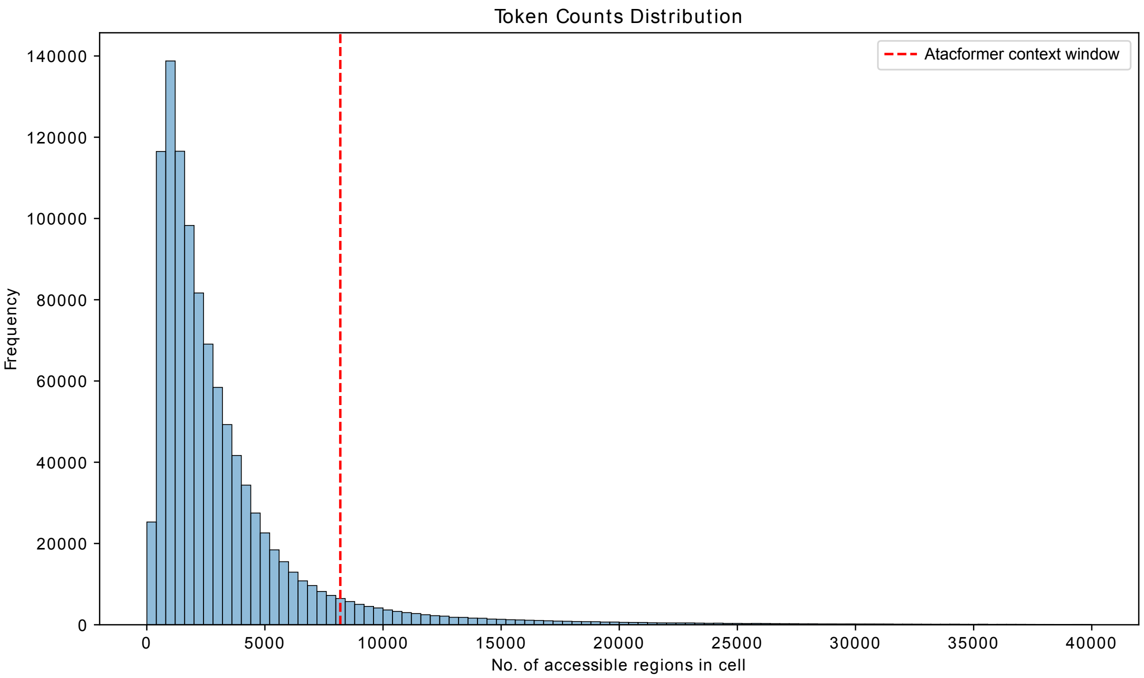

Supplemental Figure S1. Context window distribution for all cells in the pretraining corpus. *The context window is defined as the number of co-accessible regulatory elements assayed in that cell. For reference, we include the Atacformer context-window cutoff highlighting how the majority of cells are within this context window.*

bioRxiv preprint doi: https://doi.org/10.1101/2025.11.03.685753; this version posted November 4, 2025. The copyright holder for this preprint (which was not certified by peer review) is the author/funder, who has granted bioRxiv a license to display the preprint in perpetuity. It is made available under aCC-BY 4.0 International license.

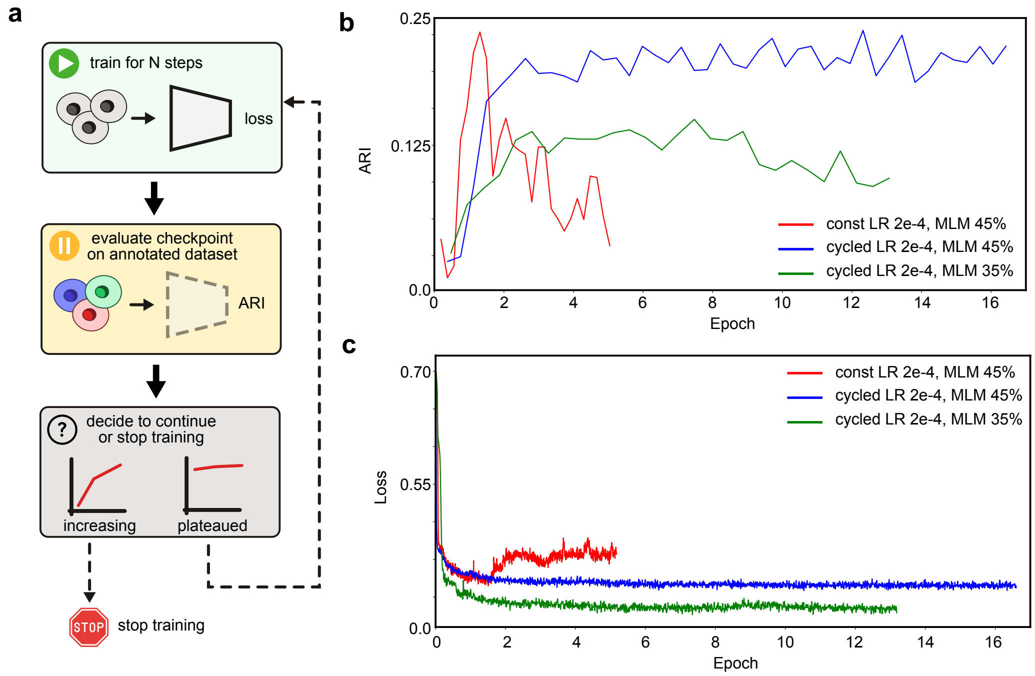

Supplemental Figure S2. ***a.*** *Schematic overview of Atacformer pre-training with monitored callbacks. We use these callbacks to determine whether to continue or stop training.* ***b.*** *Selected ARI callback curves for different learning rate schedules, MLM rates, and learning rates.* ***c.*** *Selected loss curves for different learning rate schedules, MLM rates, and learning rates. Notably, when the learning rate remains too high, the training becomes unstable, which is reflected in the loss curves, followed by a subsequent collapse in the ARI performance.*

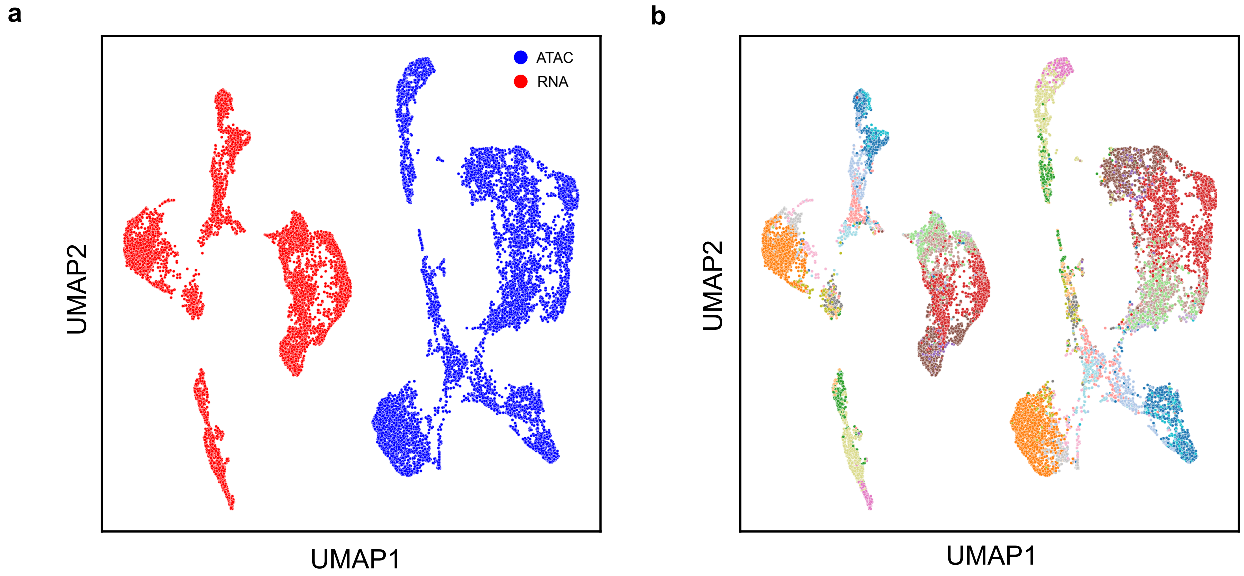

Supplemental Figure S3. Dual UMAP visualization of both the ATAC and RNA co-embeddings. a. *ATAC and RNA co-embeddings visualized in a shared UMAP space, colored by modality. The two modalities are divided along a shared axis.* ***b.*** *ATAC and RNA co-embeddings visualized in a shared UMAP space, colored by cell-type. Cell-type information is preserved.*

bioRxiv preprint doi: https://doi.org/10.1101/2025.11.03.685753; this version posted November 4, 2025. The copyright holder for this preprint (which was not certified by peer review) is the author/funder, who has granted bioRxiv a license to display the preprint in perpetuity. It is made available under aCC-BY 4.0 International license.

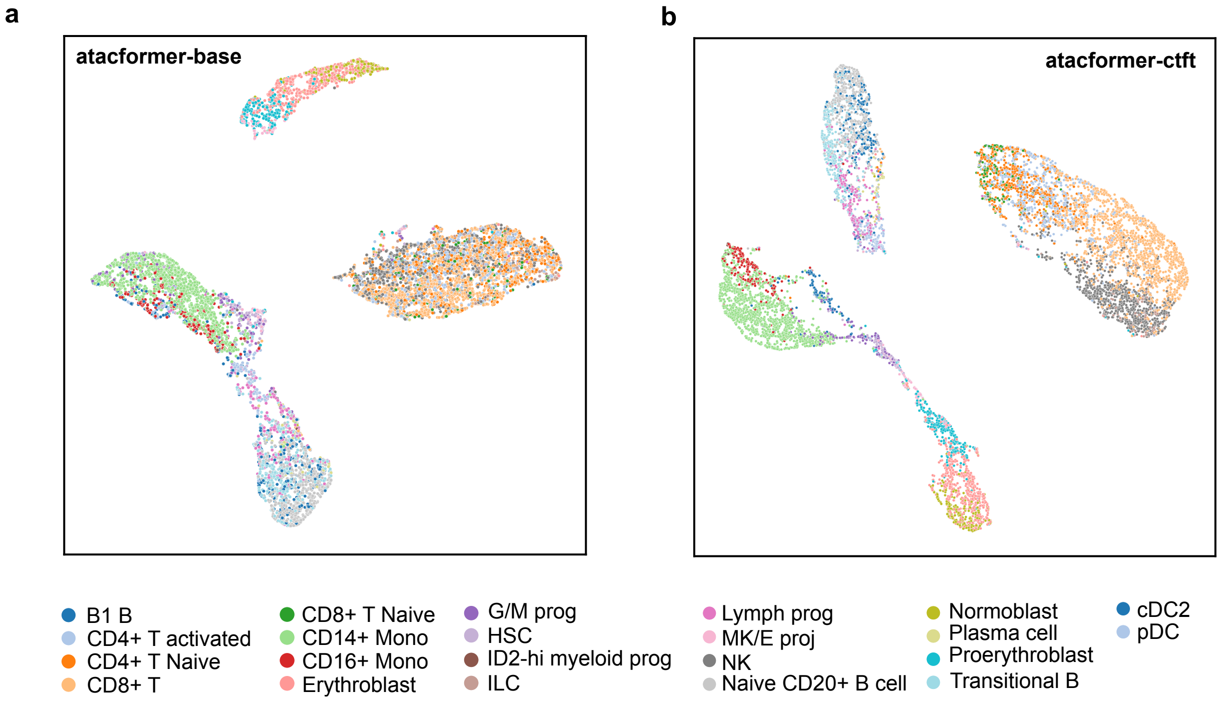

Supplemental Figure S4. *Fine-tuning Atacformer for a cell-clustering task improves latent space separation of individual cells.* ***a.*** *UMAP visualization of Luecken2021 dataset clustered using* `atacformer-base` *(before fine-tuning).* ***b.*** *UMAP visualization of Luecken2021 dataset clustered using* `atacformer-ctft` *showing a marked improvement in clustering ability.*

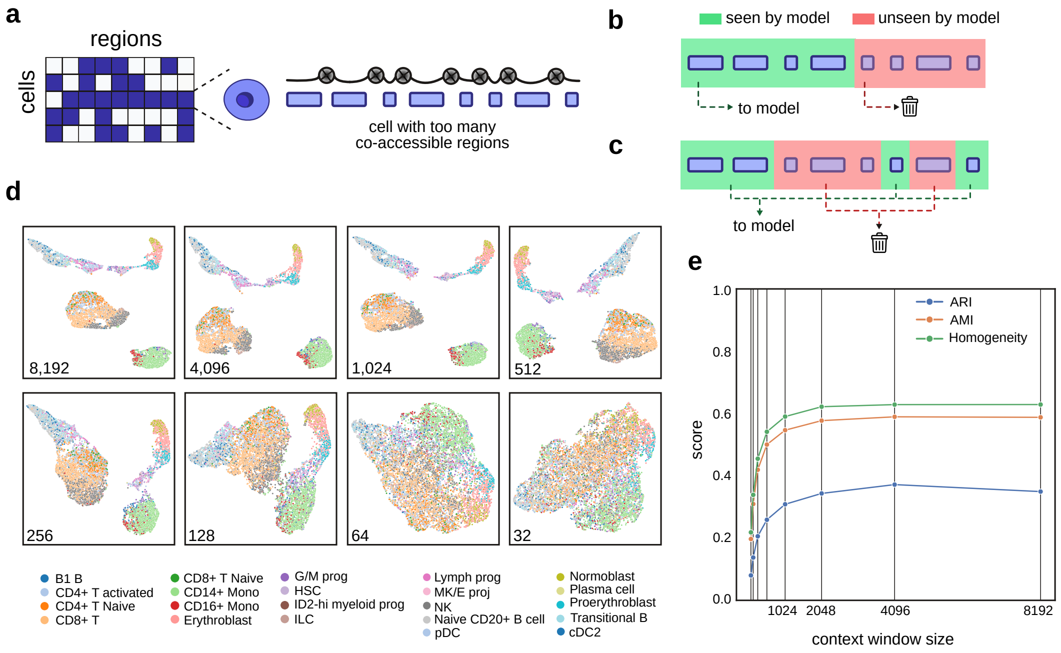

Supplemental Figure S5. Atacformer is robust to severe degradation in context-window size. a. *Schematic showing how cells are tokenized in the Atacformer framework. When the number of tokens in a cell exceeds the context window of the model, we must choose which tokens to drop before processing.* ***b.*** *Schematic of the cut-off method, where we simply keep the first C tokens in a cell, while disregarding the rest (C being the context-window size).* ***c.*** *Schematic of the random sample method, where we randomly sample C tokens from the cell, while discarding the rest.* ***d.*** *UMAP visualizations of embeddings generated from the Luecken2021 dataset using various context window sizes at inference time. A marked decrease in visually distinct clusters occurs after 512.* ***e.*** *Line plot of three clustering metrics as a function of context-window size. All plots and metrics utilized the ATAC encoder of the* `craft-100k-hg38` *model described in 2.*

bioRxiv preprint doi: https://doi.org/10.1101/2025.11.03.685753; this version posted November 4, 2025. The copyright holder for this preprint (which was not certified by peer review) is the author/funder, who has granted bioRxiv a license to display the preprint in perpetuity. It is made available under aCC-BY 4.0 International license.

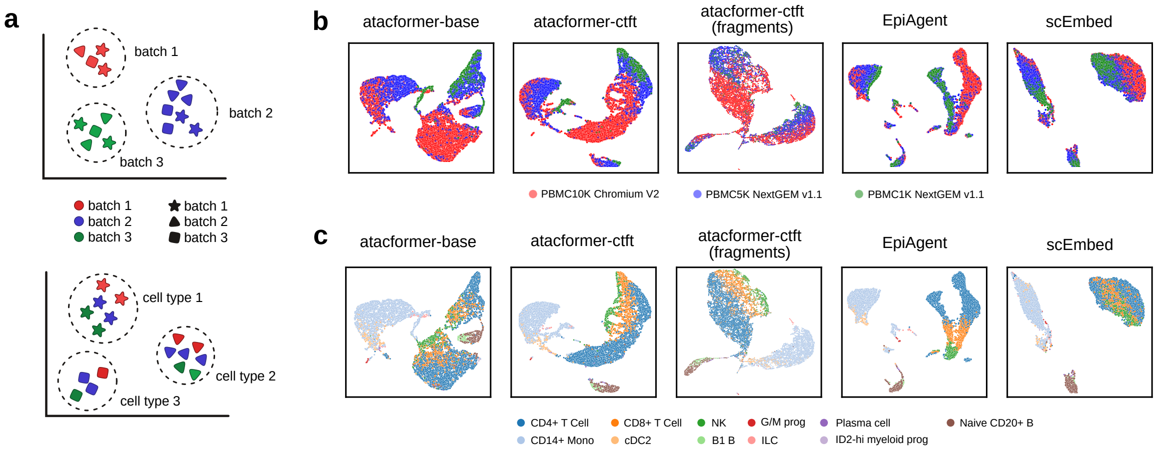

Supplemental Figure S6. Atacformer performs strong zero-shot batch correction on processed and unprocessed data. ***a.*** *Schematic overview of batch effects (top) and mitigation (bottom) when analyzing multiple datasets at once.* ***b.*** *UMAP visualizations of three PBMC dataset cell embeddings, colored by dataset. Atacformer visually exhibits equal or better clustering performance when directly producing embeddings of fragment files.* ***c.*** *UMAP visualizations of three PBMC dataset cell embeddings, colored by cell-type. Atacformer retains key biological information when directly producing embeddings of fragment files.*

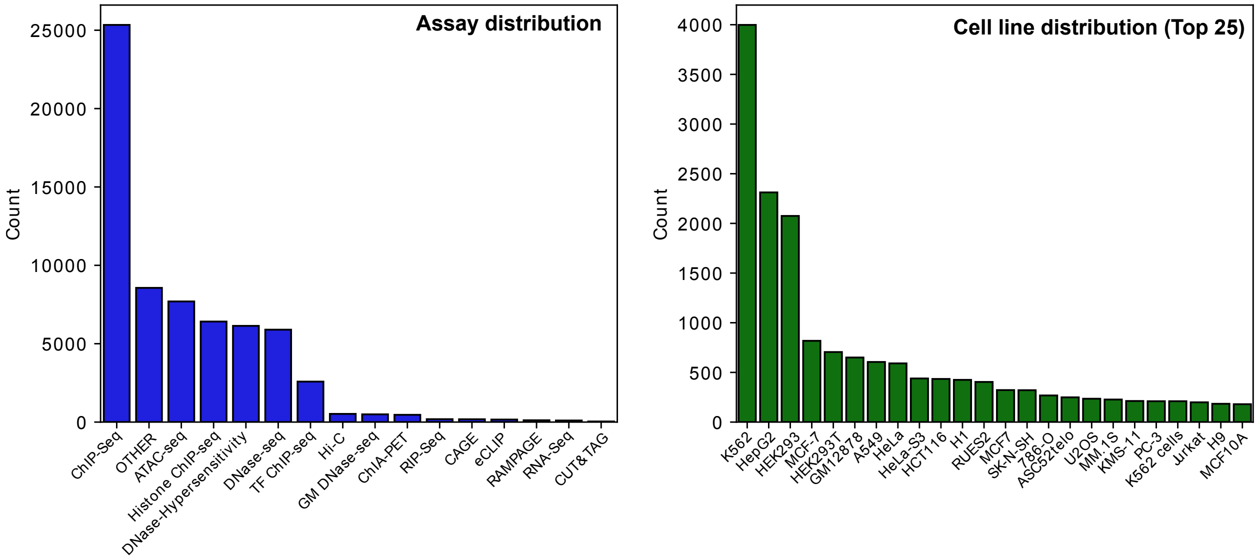

Supplemental Figure S7. Training dataset assay and cell line distribution for the bulk-ATAC model. ***a.*** *Distribution of assay types in the BEDbase bulk data training set.* ***b.*** *Distribution of the top 25 cell lines represented in the BEDbase bulk data training set.*

bioRxiv preprint doi: https://doi.org/10.1101/2025.11.03.685753; this version posted November 4, 2025. The copyright holder for this preprint (which was not certified by peer review) is the author/funder, who has granted bioRxiv a license to display the preprint in perpetuity. It is made available under aCC-BY 4.0 International license.

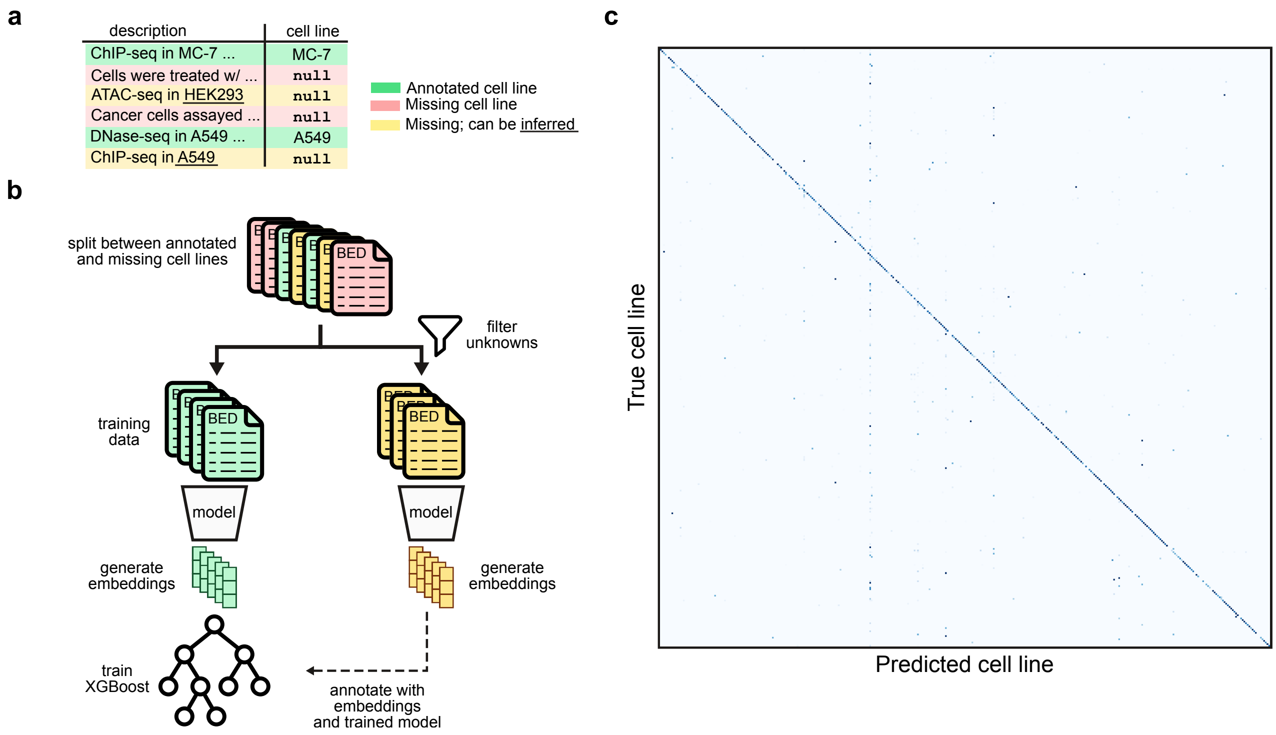

Supplemental Figure S8. Cell line imputation for missing BEDbase data using a fine-tuned Ataformer model on bulk-ATAC data. a. *Table demonstrating 3 different types of data rows: properly annotated rows, rows with missing cell-line annotation; and rows with missing but inferrable cell-line annotation.* ***b.*** *Schematic of the imputation procedure.* ***c.*** *Confusion matrix for entire cell line dataset showing broad agreement.*

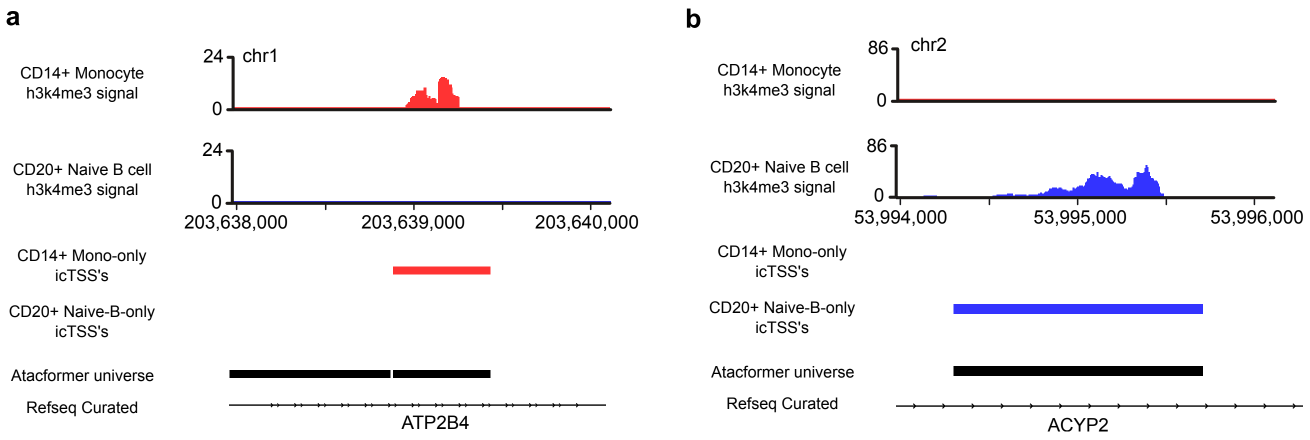

Supplemental Figure S9. Examples of H3K4me3 enrichment in icTSS regions. a. *Example monocyte-specific icTSS region showing H3K4me3 specifically in Monocytes.* ***b.*** *Example B-cell-specific icTSS showing H3K4me3 enrichment specifically in B cells.*

bioRxiv preprint doi: https://doi.org/10.1101/2025.11.03.685753; this version posted November 4, 2025. The copyright holder for this preprint (which was not certified by peer review) is the author/funder, who has granted bioRxiv a license to display the preprint in perpetuity. It is made available under aCC-BY 4.0 International license.

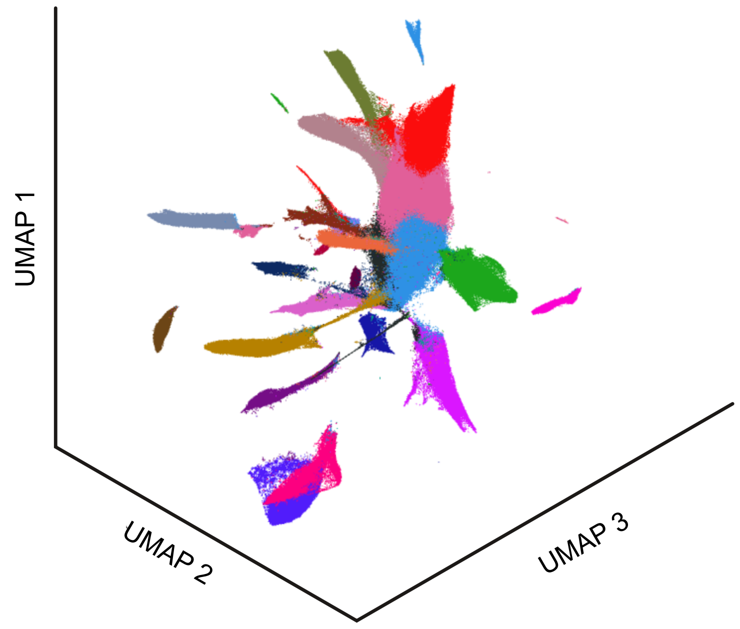

Supplemental Figure S10. Initial clustering results of the single-cell atlas from SnapATAC2. *We leverage SnapATAC2's spectral embedding methodology and cluster using Leiden clustering.*

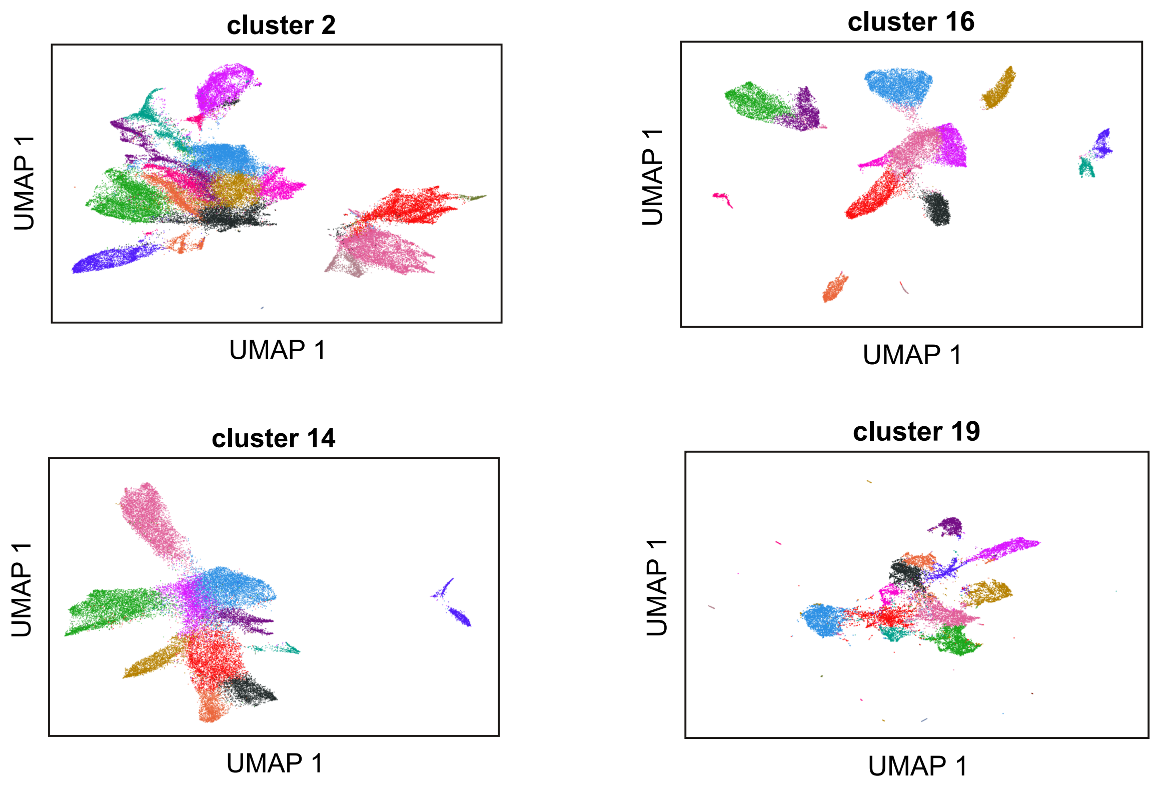

Supplemental Figure S11. Selected sub-clustering results of the single-cell atlas from SnapATAC2. *For each initial cluster, we subset the dataset and perform a secondary clustering procedure using the SnapATAC2 spectral embedding procedure. The resultant embeddings are clustered using Leiden clustering.*
